# Prognostic Significance of Sarcomatoid Differentiation in Patients With Metastatic Renal Cell Carcinoma: A Systematic Review and Meta-Analysis

**DOI:** 10.3389/fonc.2020.591001

**Published:** 2020-10-08

**Authors:** Hong Zhi, Meiling Feng, Suo Liu, Ta Na, Nandong Zhang, WuEn BiLiGe

**Affiliations:** ^1^Department of Mongolian Medicine Urology, Affiliated Hospital of Inner Mongolia University for Nationalities, Tongliao, China; ^2^Department of Mongolian Medicine Stomatology, Affiliated Hospital of Inner Mongolia University for Nationalities, Tongliao, China

**Keywords:** metastatic renal cell carcinoma, sarcomatoid differentiation, prognosis, systematic review, meta-analysis

## Abstract

**Background:**

To date, the prognostic value of sarcomatoid differentiation in patients having metastatic renal cell carcinoma (mRCC) remains inconclusive. A systematic review and meta-analysis were conducted.

**Materials and Methods:**

Relevant literatures were obtained from PubMed, Embase, and Cochrane Library published prior to May, 2020. All patients were diagnosed with mRCC and treated with surgery, cytokine therapy, targeted therapy, and immunotherapy. Sarcomatoid differentiation in the pathological specimens was identified. Each endpoint [overall survival (OS), progression-free survival (PFS), and cancer-specific survival (CSS)] was assessed using a multivariable adjusted hazard ratio (HR) and 95% confidence interval (CI).

**Results:**

Fifteen observational studies having 5,828 patients with mRCC were included. The merged results showed that patients presenting sarcomatoid differentiation had a significantly inferior OS (HR: 2.26, 95% CI: 1.82–2.81; P < 0.001), PFS (HR: 2.28, 95% CI: 1.63–3.19; P < 0.001), and CSS (HR: 2.27, 95% CI: 1.51–3.40; P < 0.001) compared to those without sarcomatoid differentiation. Subgroup analysis based on publication year, patient population, country, number of cases, and NOS score did not change the direction of results. A significant publication bias was identified for OS, but no publication bias was identified for PFS. Moreover, sensitivity analysis also verified the robustness of the results.

**Conclusion:**

This study suggested that sarcomatoid differentiation was correlated to unfavorable clinical outcomes in mRCC and may be a poor prognostic factor incorporating to prognostic models for mRCC patients.

## Introduction

Renal cell carcinoma (RCC) belongs to the most common type of kidney cancer. Though most patients with RCC are diagnosed in low-stage and can be well-managed by surgery, more than 25% of them will develop local recurrence or distant metastasis after initial treatment (1). Additionally, some patients with RCC had distant metastasis at the time of initial disease diagnosis. In the advanced stage of RCC, cases with metastatic RCC (mRCC) have a low survival rate (2). Histological prognostic roles may be helpful in treatment-choosing and survival-judging.

Sarcomatoid is the word applied to depict the morphological alterations in RCC in histology. Sarcomatoid RCC (sRCC) was initial described as an independent disease classification in 1968, with sarcomatoid components co-existing with the dominant underlying epithelial components (3). Nevertheless, by 2013, it was suggested that sarcomatoid differentiation of RCC should not be considered as an independent subtype and be classified according to its histology. When epithelial cell components cannot be identified, these tumors are classified as unclassified (4). sRCC is a rare variant of RCC and represents 1%–8% of all subtypes of RCC (5). Previous studies identified that RCC presenting sarcomatoid differentiation was correlated to a more invasive form of the disease and higher rate of metastasis, poor-response to traditional treatment, and a low overall survival (OS) rate (6, 7). Due to the unusual histology and small sample size, limited literatures have reported the prognostic value of sarcomatoid differentiation in mRCC. Moreover, inconsistent results from different studies may hinder the interpretation of this role.

Therefore, we systematically reviewed all studies about mRCC, compared the oncological outcomes between mRCC presenting and without sarcomatoid differentiation, examined the prognostic significance of sarcomatoid differentiation, and conflated the relevant results using the approach of meta-analysis.

## Materials and Methods

This study was performed in accordance with PRISMA guidelines (8) and registered on PROSPERO (CRD 42020197136).

### Literature Search

In order to obtain potential literatures, a systematic search was performed in May 2020 with three databases PubMed, Embase, and Cochrane Library. The following items were used in the search scheme: “metastatic renal cell carcinoma” (e.g., “metastatic kidney cancer”, “metastatic renal cell carcinoma”, “metastatic renal carcinoma”, and “advanced kidney cancer”), “sarcomatoid differentiation” (e.g., “sarcomatoid”, “sarcomatoid variant”, “sarcomatoid differentiation”, and “sarcomatoid feature”), and “prognosis” (e.g., “survival”, “prognosis”, “outcome”, “mortality”, “recurrence”, and “progression”). In addition, the literature references were screened manually for potential literatures. The language of relevant literatures was restricted to English.

### Inclusion and Exclusion Criteria

Two authors independently screened the literatures to identify eligible studies. Studies analyzing sarcomatoid differentiation as a prognosis predictor in cases having mRCC were included in the present study. Patients were treated with surgery, cytokine therapy, targeted therapy, and immunotherapy. The included studies had examined the prognostic significance of sarcomatoid differentiation in multivariable analyses. The following criteria were used for exclusion: 1) editorials or expert opinions, case reports, conference records, reviews, and other non-original studies; 2) basic research with tumor cell or patient samples; 3) studies included patients with non-mRCC; 4) studies did not analyze sarcomatoid differentiation as a prognosis predictor; 5) studies did not include sarcomatoid differentiation in multivariable analyses; 6) studies that were written in non-English. Moreover, in case of multiple studies based on the same cohort were retrieved, we used the most late, well-designed and complete study.

### Data Extraction and Quality Evaluations

Two authors independently evaluate the quality of included literatures and extracted the required data. The primary outcome was OS, the secondary outcomes embraced progression-free survival (PFS) and cancer-specific survival (CSS). A pre-designed table was used to extract data, the items included first author’s name, publication year, study design, patient source and country, study period, sample size, patients’ age, histology, therapy, follow-up duration, and adjusted variables in multivariable analyses. In addition, the hazard ratios (HRs) and 95% conﬁdence intervals (CIs) of sarcomatoid differentiation for each endpoint in multivariable analyses were extracted.

The Newcastle-Ottawa Scale was used to evaluate the methodological quality of each study (9). According to three important domains, each study was scored from 0 to 9, we only included studies with score 6 or higher.

### Statistical Analysis

At first, we analyzed the three outcomes OS, PFS, and CSS with all included studies. After that, subgroup analyses were performed for OS and PFS, the classification variables had year, patient source, patient region, number of cases, and NOS score. The statistical process was conducted with Stata 12.0 software (StatCorp, College Station, TX, USA). Statistical heterogeneity among included studies was assessed with Cochran’s Q test and *I^2^* statistic. When p < 0.05 or *I^2^* > 50%, which indicates that significant heterogeneity existed among studies, the random-effect model was used for meta-analyses; otherwise, the fixed-effect model was used. Each endpoint was assessed using a multivariable adjusted HRs and 95% confidence interval (CI). Meta-regression analysis was performed to precisely evaluate heterogeneity. Funnel plots and Begg’s/Egger’s tests were applied to assess publication bias. Additionally, sensitivity analyses were conducted to estimate the robustness of the results. A two-tailed p value < 0.05 was deemed as statistically significant.

## Results

### Literature Search

**Figure 1:**
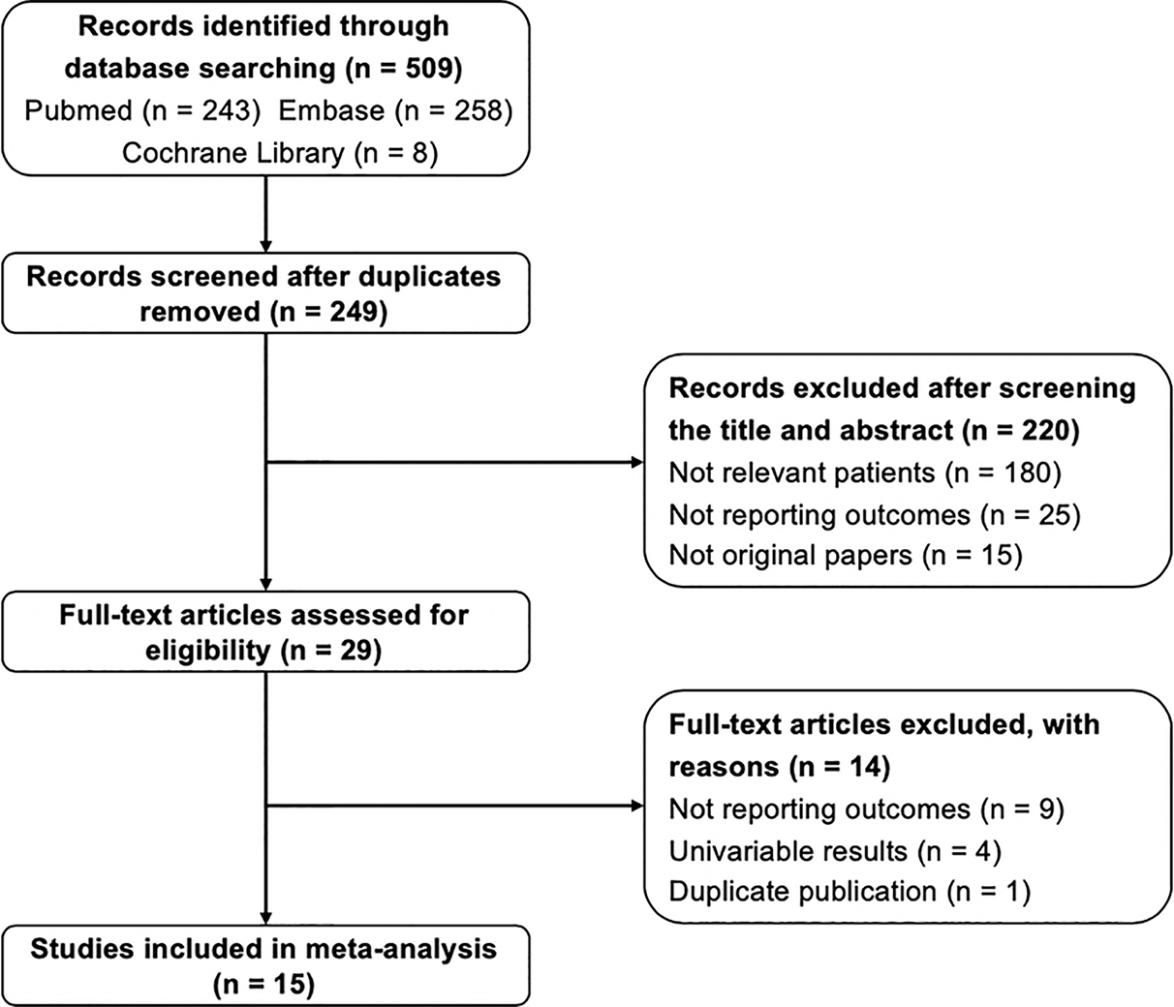
Flow chart of literature search and selection process.

The flowchart of study selection process was presented in **Figure 1**. After the initial search of three databases, 509 records were obtained. After removing duplicates, 249 records were screened. According to the title and abstract screening, 220 records have been excluded due to not relevant patients, not reporting outcomes, and non-original articles. The full-text of 29 studies were evaluated, 9 of them were excluded because of not reporting outcomes, 4 of them due to without multivariable results, 1 of them was duplicate publication. Finally, 15 studies in accordance with the inclusion criteria were included in the present study.

### Study Characteristics

Baseline features, outcomes, and adjusted variables of the 15 eligible literatures were shown in **Tables 1**, **2**. These literatures enrolled 5,828 patients (range from 35 to 2,286), with a mean or median age ranging from 53 to 75 years, with a median or mean follow-up duration ranging from 7 to 52.7 months. Except for one study was prospectively designed, all the remaining studies were retrospectively designed. Nine and six studies respectively analyzed single-institution and multi-institution cohort. For all studies, three were performed in USA, three in Japan, three in Korea, two in China, two in Europe, one in UK, and one in multiple countries. Patients were treated with surgery, cytokine therapy, targeted therapy, and immunotherapy. Multiple therapies were applied in most patients with various pathologic types of RCC. OS and PFS were the most assessed outcomes; hematological and clinicopathological parameters were mainly adjusted in the multivariable analyses.

**Table 1:** Baseline characteristics of included studies.

First author	Year	Design	Patient population	Study period	Country	Sample size	Age (years)	Histology	TNM stage	NOS score
Takagi	2020	Retro	Single institution	2008–2018	Japan	51	65 (57–71)	All	T1-3NXM1	7
Luzzago	2020	Retro	Multi-institution	2006–2015	USA	1573	62.6 mean	Non-clear cell	T1-4N0-XM1	7
Verbiest	2019	Pro	Multi-institution	–	Europe	366	63 median	Clear cell	T1-4NXM1	7
Uccello	2019	Retro	Single institution	2012–2018	UK	35	75 (70–91) R	All	–	7
Takeshita	2019	Retro	Single institution	1988–2017	Japan	50	60 (11–82) R	All	–	6
Fukuda	2018	Retro	Single institution	1984–2015	Japan	170	63.5 (61.4–64.5)	All	T1-4N0-2M1	8
Han	2017	Retro	Single institution	2005–2015	Korea	101	58.4 ± 11.4	All	T1-3NXM1	8
Gu	2017	Retro	Single institution	2006–2014	China	184	54.3 (13.0)	All	–	7
Choi	2017	Retro	Single institution	1990–2015	Korea	93	53 median	All	T1-4N0-2M1	7
Abel	2017	Retro	Multi-institution	2000–2014	USA	427	61.5 (54.4–69.8)	All	T3-4NXM1	7
Kara	2016	Retro	Single institution	2005–2013	USA	118	–	All	T1-4N0-XM1	7
Yu	2015	Retro	Multi-institution	2007–2014	China	140	57 (17–79) R	All	–	7
Kyriakopoulos	2015	Retro	Multi-institution	2008–2013	Multiple	2286	58 mean	All	–	8
Tosco	2013	Retro	Multi-institution	1998–2011	Europe	109	62 (25–82) R	All	T1-4NXM1	8
Kwak	2007	Retro	Single institution	1990–2004	Korea	125	58 (20–79) R	All	T1-4NXM1	7

Retro, retrospective; Pro, prospective; R, range; NOS, Newcastle-Ottawa Scale.

**Table 2:** Follow-up and oncological outcomes.

First author	Year	Treatment	Follow-up duration, mon	Outcomes	Adjusted factors
Takagi	2020	Nephrectomy or partial nephrectomy for primary lesion, metastasectomy	49 median	PFS	Metastatic sites
Luzzago	2020	Cytoreductive nephrectomy, systemic therapy, combination of cytoreductive nephrectomy and systemic therapy, or no treatment	7 (3–13)	OS	Treatment modality, age, gender, race, marital status, socioeconomic status, year of diagnosis, size, T stage, N stage, metastasectomy
Verbiest	2019	Systemic therapy or metastasectomy	–	PFS, OS	IMDC risk group
Uccello	2019	First-line pazopanib or sunitinib	33.4 median	OS	Karnofsky performance status, absolute neutrophil count, hypertension
Takeshita	2019	Surgery, radiation therapy, cytokine therapy, targeted therapy, immunotherapy	8.2 (5.5–13.7)	OS	Graded prognostic assessment score, histology, local therapy for brain metastasis
Fukuda	2018	Cytoreductive nephrectomy, targeted therapy, cytokine therapy, metastasectomy, radiation therapy	52.4 median	OS	ECOG-PS, MSKCC risk, histology, clinical T stage, primary tumor size, number of metastatic organs, non-regional lymph node metastasis, liver metastasis, Glasgow prognostic score
Han	2017	Nephrectomy or partial nephrectomy for primary lesion, metastasectomy, targeted therapy	37.0 (18.3–59.4)	PFS, OS	Fuhrman grade, metastasectomy, metastatic sites, time to metastasis, corrected calcium, first metastasis site, hemoglobin
Gu	2017	Cytoreductive nephrectomy, targeted therapy, cytokine therapy	23.3 (14.6)	PFS, OS	Tumor site, tumor size, histology, fuhrman grade, tumor necrosis, number of metastatic site, neutrophilia, anemia thrombocytosis, lymphovascular invasion
Choi	2017	Gamma Knife radiosurgery, radiation therapy, neurosurgery, targeted therapy, immunotherapy	44.2 (22.6–88.2)	PFS, OS	Brain metastasis type, bone metastasis at brain metastasis diagnosis, number of brain metastasis, MSKCC risk group, local therapy for brain metastasis, systemic therapy for brain metastasis
Abel	2017	Cytoreductive nephrectomy, lymphadenopathy	18.9 (6.8–43.9)	OS	Surgery to systemic therapy, hemoglobin, corrected serum calcium, serum lactate dehydrogenase, absolute platelet count, serum albumin, retroperitoneal lymphadenopathy, systemic symptoms present, thrombus level
Kara	2016	Cytoreductive nephrectomy	–	CSS	Node involvement, ECOG performance status
Yu	2015	Cytoreductive nephrectomy, targeted therapy, cytokine therapy	24 (3–88) R	PFS, OS	Pathology, progressive disease, gender, prior nephrectomy, prior systemic therapy, multi-organs, metastasis, having at least once ADEs with grade 3 or 4
Kyriakopoulos	2015	Targeted therapy	–	PFS, OS	Karnofsky performance status, diagnosis to treatment interval, calcium, hemoglobin, neutrophilia, thrombocytosis
Tosco	2013	Radical nephrectomy or partial nephrectomy, metastasectomy, immunotherapy, cytokines, targeted therapy, and radiotherapy, chemotherapy	52.7 (1.37–283) R	CSS	T stage, fuhrman grade, ECOG performance status, disease-free interval, nonpulmonary metastasis, multiorgan metastasis, targeted therapy, synchronous metastasis, resection margins
Kwak	2007	Cytoreductive nephrectomy, metastasectomy, cytokine therapy	17.4 (2.4–78.9)	PFS, OS	Age, sex, ECOG performance status, T stage, histologic type, fuhrman’s grade, metastasis sites, time to metastasis, metastasectomy, immunotherapy regimen

PFS, progression-free survival; OS, overall survival; CSS, cancer-specific survival; R, range.

### Meta-Analysis Results

**Figure 2:**
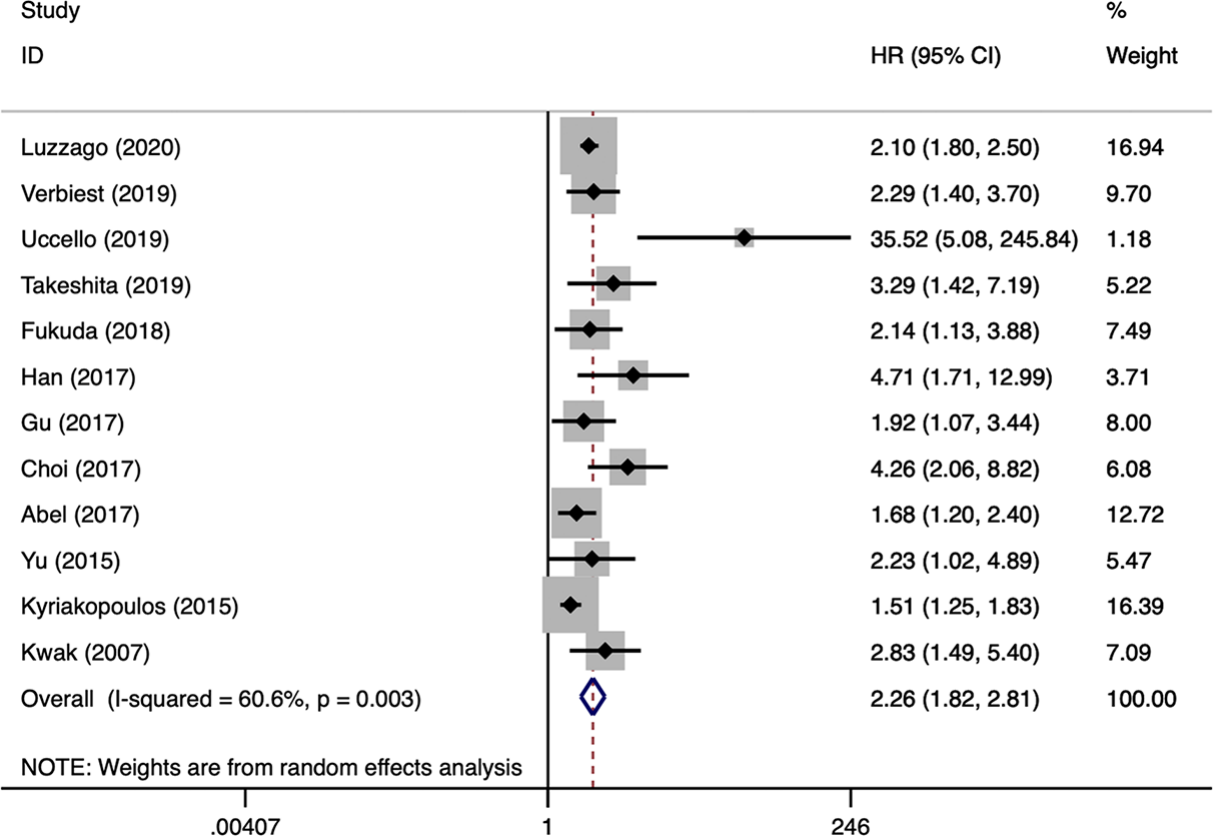
Forest plots of pooled hazard ratios for overall survival.

**Figure 3:**
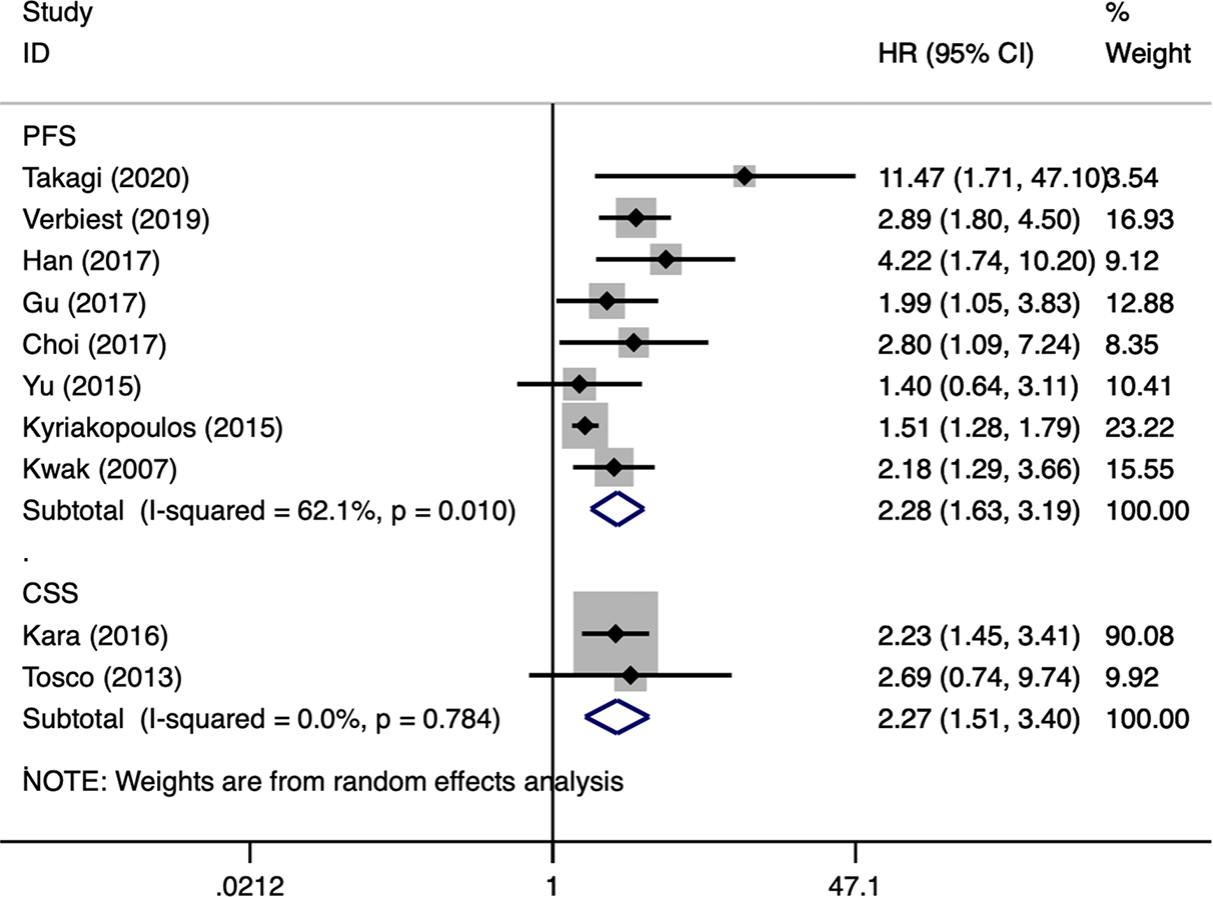
Forest plots of pooled hazard ratios for progression-free survival and cancer-specific survival.

OS was the most assessed outcome, which was reported by 12 studies (10–21). Because of significant inter-study heterogeneity (*I^2^* = 60.6%, P = 0.003), a random-effect model was applied. The pooled results indicated that sarcomatoid differentiation in mRCC was associated with a poor OS (HR: 2.26, 95% CI: 1.82–2.81; P < 0.001) (**Figure 2**). Meta-regression analysis and subgroup analyses were performed to precisely evaluate heterogeneity. When the studies were stratified based on publication year, number of institutions, patient country, number of patients, NOS score, sarcomatoid differentiation was also found to be a significant prognostic role of OS for patients with mRCC. Number of patients perhaps to be the reason of heterogeneity, however, the other variables was not (**Table 3**). PFS was the secondary outcome, which was reported by eight studies (11, 15–17, 19–22). Because of significant inter-study heterogeneity (*I^2^* = 62.1%, P = 0.010), a random-effect model was applied. The pooled results indicated that sarcomatoid differentiation in mRCC was correlated to a poor PFS (HR: 2.28, 95% CI: 1.63–3.19; P < 0.001) (**Figure 3**). When the studies were stratified based on publication year, number of institutions, patient country, number of patients, sarcomatoid differentiation was also found to be a significant prognostic role of PFS for patients with mRCC. Publication year perhaps to be the reason of heterogeneity, however, the other variables were not (**Table 3**). Two studies reported the results of CSS (23, 24). The pooled results showed that sarcomatoid differentiation in mRCC was correlated to an inferior CSS (HR: 2.27, 95% CI: 1.51–3.40; P < 0.001) (**Figure 3**).

**Table 3:** Subgroup analyses for overall survival and progression-free survival.

Subgroup	Studies	HR (95% CI)	P value	Meta-regressionP value	Heterogeneity	
					*I^2^* (%)	P value
*Overall survival*						
Year of publication				0.590		
2018–2020	5	2.53 (1.73–3.69)	<0.001		56.5	0.056
2007–2017	7	2.16 (1.60–2.91)	<0.001		58.4	0.025
Patient population				0.051		
Single institution	7	2.90 (2.19–3.84)	<0.001		47.1	0.078
Multi-institution	5	1.84 (1.64–2.05)	<0.001		49.4	0.095
Region				0.223		
Asia	7	2.68 (2.05–3.50)	<0.001		0.0	0.540
Non-Asia	4	2.21 (1.51–3.22)	<0.001		69.4	0.020
Sample size				0.007		
<130	5	3.85 (2.64–5.61)	<0.001		36.4	0.179
>130	7	1.85 (1.66–2.06)	<0.001		26.4	0.227
NOS score				0.401		
<=7	9	2.16 (1.90–2.46)	<0.001		48.1	0.051
>7	3	2.08 (1.21–3.57)	<0.001		64.2	0.061
*Progression-free survival*						
Year of publication				0.016		
2017-2020	5	2.91 (2.12–4.00)	<0.001		13.5	0.328
2007-2015	3	1.55 (1.33–1.82)	<0.001		0.0	0.400
Patient population				0.244		
Single institution	5	2.59 (1.85–3.62)	<0.001		25.1	0.254
Multi-institution	3	1.85 (1.16–2.94)	0.010		71.2	0.031
Region				0.784		
Korea	3	2.62 (1.75–3.93)	<0.001		0.0	0.447
Non-Korea	4	2.45 (1.44–4.17)	0.001		50.8	0.107
Sample size				0.157		
<130	4	2.85 (1.92–4.22)	<0.001		33.1	0.213
>130	4	1.86 (1.30–2.67)	0.001		59.0	0.062

HR, hazard ratio; CI, confidence interval; NOS, Newcastle-Ottawa Scale.

### Publication Bias and Sensitivity Analysis

**Figure 4:**
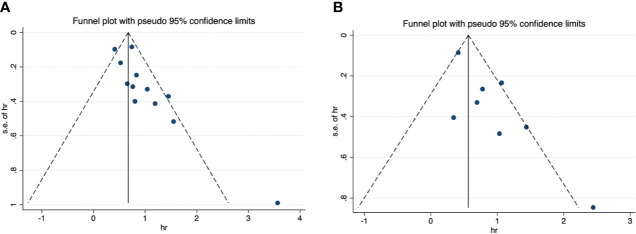
Funnel plots of overall survival and progression-free survival. **(A)** overall survival; **(B)** progression-free survival.

**Figure 5:**
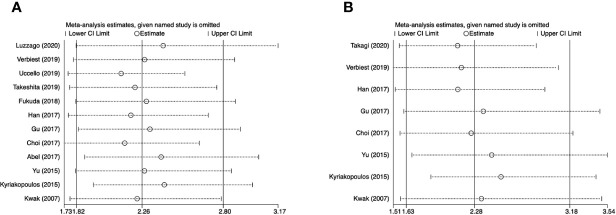
Sensitivity analyses of overall survival and progression-free survival. **(A)** overall survival; **(B)** progression-free survival.

For OS, the funnel plot seems to be asymmetric (**Figure 4A**), the p-values of the Begg’s and Egger’s tests were both lower than 0.05 (P-Begg = 0.007; P-Egger = 0.012). However, the funnel plot indicated that PFS had no evident of asymmetry (**Figure 4B**), the p-values of the Begg’s and Egger’s tests were both higher than 0.05 (P-Begg = 0.386; P-Egger = 0.115). In sensitivity analysis, the merged HR for OS ranged from 2.12 (95% CI: 1.76–2.55) to 2.41 (95% CI: 1.93–3.00) (**Figure 5A**). Likewise, the merged HR for PFS ranged from 2.12 (95% CI: 1.56–2.86) to 2.53 (95% CI: 1.86–3.43) (**Figure 5B**). The findings showed that the results are robust and reliable.

## Discussion

Sarcomatoid differentiation is considered an undesirable parameter from a prognostic point of view and is correlated to the adverse clinical features and biological aggressiveness of RCC that lead to unfavorable survival and reduced response to large systemic therapies (25, 26). To this day, multiple literatures have investigated the prognostic significance of sarcomatoid differentiation in cases with RCC. However, inconsistent results from different studies may hinder the interpretation of this role.

Hence, by assessing oncological outcomes, Zhang et al. (27) have performed a systematic review and meta-analysis to assess the pathologic and prognostic effects of sarcomatoid differentiation on cases having RCC. They have found that sarcomatoid differentiation was closely correlated with inferior long-term outcome and was significantly associated with high TNM staging and Fuhrman grade, positive lymph node, mixed histologic type. This comprehensive study including 35 literatures was well-preformed and firstly examined the prognostic significance of sarcomatoid differentiation in RCC. Nevertheless, they have analyzed metastatic and non-metastatic renal tumor together. To our best knowledge, the two stages or forms of RCC have very discrepant features and survival, and it was improper to combine the data. In addition, literatures with univariate analysis data may bring about potential confounders and selection bias. Considering many of the latest literature, we used the results of multivariate analysis to investigate the prognostic significance of sarcomatoid differentiation in mRCC.

With the data from 15 literatures, the present meta-analysis represents the most integrated study to systematically examine the prognostic significance of sarcomatoid differentiation in mRCC patients. The pooled results indicated that sarcomatoid differentiation was significantly correlated to unfavorable OS (HR: 2.26, 95% CI: 1.82-2.81; P < 0.001), PFS (HR: 2.28, 95% CI: 1.63-3.19; P < 0.001) and CSS (HR: 2.27, 95% CI: 1.51–3.40; P < 0.001) in patients with RCC. Moreover, according to subgroup analyses of several baseline characteristics, sarcomatoid differentiation was still an independent predictor of prognosis for mRCC. The robustness of the results was further verified by sensitivity analysis. Presently, for patients with mRCC, a number of prognostic models have been designed to help the selection of appropriate therapies and to predict tumor outcomes in individual patients (28). Most of risk factors in them are clinical characteristics and blood biomarkers, incorporating histological variables like sarcomatoid differentiation in these models may improve the accuracy of prediction.

RCC presenting sarcomatoid differentiation are more malignant, therefore require more aggressive treatment. Although some progress has been made in the treatment of mRCC in recent years, clinical data are mainly derived from patients with clear cell RCC (29). The lack of clinical evidence for sRCC limits our understanding of the efficacy and safety of this variant. Therefore, many management recommendations are extrapolated from the available evidence at the clear cell RCC. Data on sRCC is lacking, however, some studies have shown that the use of anti-VEGF targeted therapy was not effective (30, 31). Phase III studies using first-line immune-checkpoint inhibitors to treat mRCC showed high PD-L1 expression in the sRCC subgroup (32). Subgroup analysis of IMmotion151 trial found that patients with sRCC receiving the combination therapy of atezolizumab and bevacizumab experienced favorable PFS when compared with those receiving sunitinib (33). In addition, the CheckMate214 trial identified that nivolumab and ipilimumab had a satisfactory response rate (57%) to sRCC. Based on the published randomized clinical trials, a meta-analysis by Iacovelli et al. (34) found that patients with sRCC receiving immune checkpoint inhibitor-based combinations experienced improved PFS and OS, achieved higher objective response rate and complete responses compared with sunitinib, which may redefine the first line treatment for sRCC. Presently, several studies have investigated the genomic features of RCC with sarcomatoid differentiation. Malouf et al. (35) found that sarcomatoid and clear cell RCC had different driver mutations. The sarcomatoid and epithelial components of sRCC have similar genomic features. In the light of the presence of TP53 or NF2 mutations, sRCC could be divided into two categories. Ito et al. (36) found that a sarcomatoid element in RCC was correlated to higher rates of chromosome imbalance. Bi et al. (37) identified that sarcomatoid elements showed more special somatic mutations, notably in cancer-driver genes. These findings may have important meanings for understanding the tumorigenesis and enhanced aggressiveness of sRCC and for perfecting systematic treatment regimens.

There are several limitations for the present study need to be noted. First, most included studies were retrospectively designed to analyze patients from single institution. Though only results from multivariable analyses were included, the potential confounders and selection bias were inevitable. Second, significant heterogeneity was found among studies for OS and PFS, which may cause by difference in study design, patients’ characteristics, treatment strategy, and so on. Third, there were inconsistencies in the criteria for sarcomatoid differentiation in the pathological specimens, which may lead to potential bias. Therefore, the diagnosis of sarcomatoid differentiation should follow strict morphological criteria. Fourth, several studies have examined the prognostic role of sarcomatoid and rhabdoid differentiation in RCC patients. Rhabdoid differentiation in RCC refers to the development of tumor cells that are morphologically similar to rhabdoid cells but differ in their ultrastructural characteristics and immunophenotypes. Similar to sarcomatoid differentiation, rhabdoid differentiation of RCC is considered a predictor of poor prognosis. Existing studies supported sarcomatoid differentiation and/or rhabdoid differentiation in inclusion of grade 4 RCC. However, in assessing the prognosis of renal cancer, it seems inappropriate to treat sarcomatoid differentiation and rhabdoid differentiation equally. In addition, a publication bias was identified in OS, thus overestimating the association between sarcomatoid differentiation and overall mortality risk.

Despite the above limitations, this study is the most comprehensive and up-to-date study on this subject. Our findings advised that sarcomatoid differentiation was correlated to inferior OS, PFS and CSS for patients with mRCC after adjusting for common variables. These results showed that sarcomatoid differentiation can be a potentially poor prognosis predictor that can be used to classify risk stratification and incorporated to existed prognostic models. Given the limitations of the current study, multi-institution studies applying standardized criteria and methods are needed to confirm the prognostic significance of sarcomatoid differentiation in mRCC.

## Data Availability Statement

The original contributions presented in the study are included in the article/supplementary material; further inquiries can be directed to the corresponding author.

## Author Contributions

Conception and design: HZ and MF. Data collection or management: HZ, SL, and TN. Data analysis: HZ, NZ, and WB, Manuscript writing/editing: HZ and MF. All authors contributed to the article and approved the submitted version.

## Conflict of Interest

The authors declare that the research was conducted in the absence of any commercial or financial relationships that could be construed as a potential conflict of interest.
